# ENABLE 2017, the First European PhD and Post-Doc Symposium. Session 1: Building the Foundations of Biology: Synthetic and Cellular Research

**DOI:** 10.3390/biom8030046

**Published:** 2018-07-06

**Authors:** Gianmarco Di Mauro, Ambra Dondi, Giovanni Giangreco, Alexander Hogrebe, Elja Louer, Elisa Magistrati, Meeli Mullari, Gemma Turon, Wouter Verdurmen, Helena Xicoy Cortada, Sanja Zivanovic

**Affiliations:** 1 Institute for Research in Biomedicine (IRB Barcelona), The Barcelona Institute of Science and Technology, Baldiri Reixac 10, 08028 Barcelona, Spain; gemma.turon@irbbarcelona.org (G.T.); sanja.zivanovic@irbbarcelona.org (S.Z.); 2 European School of Molecular Medicine (SEMM), via Adamello 16, 20139 Milano, Italy; ambra.dondi@ieo.it (A.D.); giovanni.giangreco@ieo.it (G.G.); elisa.magistrati@ifom.eu (E.M.); 3 Novo Nordisk Foundation Center for Protein Research, University of Copenhagen, Blegdamsvej 3B, DK-2200 Copenhagen N, Denmark; alexander.hogrebe@cpr.ku.dk (A.H.); meeli.mullari@cpr.ku.dk (M.M.); 4 Radboud Institute for Molecular Life Sciences (RIMLS), Radboud University Medical Center, Geert Grooteplein 28, 6525 GA Nijmegen, The Netherlands; Elja.Louer@radboudumc.nl (E.L.); Wouter.Verdurmen@radboudumc.nl (W.V.); Helena.Xicoy@radboudumc.nl (H.X.C.)

**Keywords:** biomedicine, symposium, synthetic biology, super resolution microscopy, stem cells, cancer, protocells, morphogenesis

## Abstract

The European Academy for Biomedical Science (ENABLE) is an initiative funded by the European Union Horizon 2020 program involving four renowned European Research Institutes (Institute for Research in Biomedicine—IRB Barcelona, Spain; Radboud Institute for Molecular Life Sciences—RIMLS, the Netherlands; Novo Nordisk Foundation Center for Protein Research—NNF CPR, Denmark; European School of Molecular Medicine—SEMM, Italy) and an innovative science communication agency (Scienseed). With the aim of promoting biomedical science of excellence in Europe, ENABLE organizes an annual three-day international event. This gathering includes a top-level scientific symposium bringing together leading scientists, PhD students, and post-doctoral fellows; career development activities supporting the progression of young researchers and fostering discussion about opportunities beyond the bench; and outreach activities stimulating the interaction between science and society. The first European PhD and Postdoc Symposium, entitled “Breaking Down Complexity: Innovative Models and Techniques in Biomedicine”, was hosted by the vibrant city of Barcelona. The scientific program of the conference was focused on the most recent advances and applications of modern techniques and models in biomedical research and covered a wide range of topics, from synthetic biology to translational medicine. Overall, the event was a great success, with more than 200 attendees from all over Europe actively participating in the symposium by presenting their research and exchanging ideas with their peers and world-renowned scientists.

## 1. Introduction

**Figure 1:**
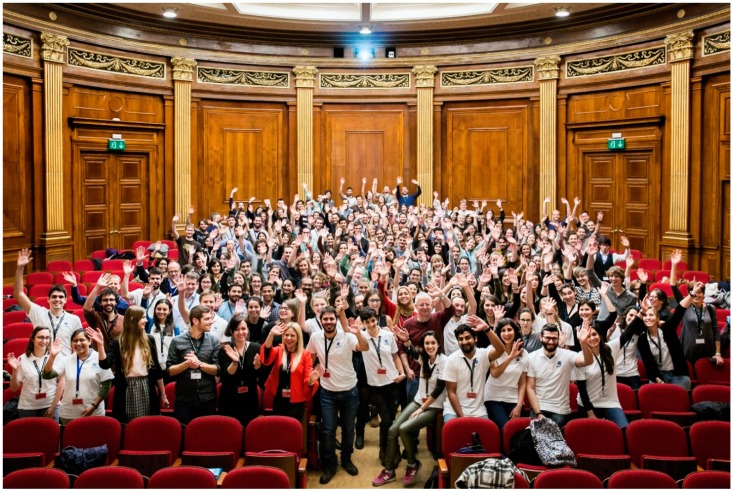
Attendees at ENABLE 2017.

Funded by Horizon 2020, the European Academy for Biomedical Science (ENABLE) project celebrates European research and brings together PhDs and postdocs from all over Europe via activities organized by volunteers and coordinators from the four host institutes—Institute for Research in Biomedicine (IRB) in Barcelona, European School of Molecular Medicine (SEMM) in Milan, Radboud Institute for Molecular Life Sciences (RIMLS) in Nijmegen, and Center for Protein Research (CPR) in Copenhagen. In November 2017, the first ENABLE conference took place in Barcelona, Spain. The organization of this conference started almost two years earlier and involved a group of 35 volunteer PhD students and postdocs from the four aforementioned research institutes and the support of institute coordinators and the innovative science communication agency, Scienseed. Like many young scientists nowadays, we felt isolated in our own research areas and wanted to build networks beyond our own fields. This is why we launched ENABLE and what the first ENABLE conference also achieved: it involved young scientists opening the academic world from within and promoted crosstalk between disciplines, collaboration with industry, and communication with society at large. The conference in Barcelona was a huge success, with the participation of 272 young researchers from more than 25 countries within the European Union (EU) and beyond (Figure 1). Companies were also thrilled by our approach, as reflected by our 10 sponsors, who provided more than 60 travel grants, and the more than 20 organizations that were present at our Career Day.

The scientific part of the ENABLE conference was a symposium entitled “Breaking Down Complexity: Innovative Models and Techniques in Biomedicine”. It comprised four sessions, spanning from molecular research to clinical research and potential new therapies. In each session, two distinguished keynote speakers presented their research and gave an overview of their work and the directions their fields are taking. Each keynote lecture was followed by two outstanding presentations by postdocs or PhDs chosen from among the 272 participants. In addition, 100 posters from a range of biomedical fields were presented by the participants, thus facilitating additional discussion between young researchers with diverse backgrounds. Last but not least, the conference also drew society into the discussion by organizing public debates with experts in the field, inviting school children to take part in scientific problem-solving activities at IRB Barcelona, and giving 24 participating young scientists the opportunity to present their work to the public.

## 2. Session 1: Building the Foundations of Biology: Synthetic and Cellular Research

**Dr. Martin Hanczyc** (Principal Investigator, Centre for Integrative Biology, University of Trento, Trento, Italy) opened the Symposium with a keynote lecture entitled “Droplet-based protocell models with embedded metabolism”. His research is aimed at integrating various aspects of artificial, synthetic, and natural life, in an attempt to assess the boundaries between living and non-living organisms. In particular, Prof. Hanczyc’s lab is currently involved in: (1) developing strategies to create artificial cells, taking advantage of lipid bilayer interfaces and droplet-based emulsions; (2) developing robot interfaces to improve treatment and clean-up of wastewater for energy generation; (3) using polymers of hydrogen cyanide to mimic and understand early prebiotic chemistry on Earth. Prof. Hanczyc explained that his research focuses on understanding the fundamental principles of living and evolving systems through experimental science. Prof. Hanczyc builds synthetic systems where dynamic life-like properties emerge when self-assembled systems are pushed away from equilibrium. He uses an experimental model of bottom-up synthetic biology: droplet-based protocol with chemically active oil droplets. The droplet-based protocol (oil and water) was first used in 1892 by a zoologist that found similarities between the motility of the droplets with amoeba movement. Chemically speaking, the environment can move things: for example, changes in pH result in alterations in interfacial surface properties. One of the systems used by Hanczyc is chemical chemotaxis with salt concentration gradients and decanol.

Next, **Dr. Anne-Sophie Huart** (Post-Doctoral researcher, European Molecular Biology Laboratory (EMBL, Hamburg and Heidelberg, Germany) introduced her work with a short talk entitled “Molecular mechanisms behind DAPK regulation: how phosphorylation switches work”. Death-Associated Protein Kinases (DAPKs) belong to the superfamily of Ca^2+^/calmodulin (CaM)-dependent kinases and are involved in the regulation of the signaling pathways leading to apoptosis or autophagy. DAPK loss- and gain-of-function have been associated with various cancer and neurodegenerative diseases. The study presented during the talk focused on the regulation of DAPK1 and DAPK2 activity by their autoregulatory domain. In particular, it explored the effect of their phosphorylation as well as interaction with the regulatory CaM protein on DAPK structural dynamics. The results shown demonstrate that CaM activates the kinase activity of DAPKs by displacing their autoregulatory domain away from the catalytic domain and thus making the substrate-binding pocket of the kinase available. Furthermore, they show that while DAPK autophosporylation works as a molecular switch that is able to lock DAPK in a closed conformation with low affinity for CaM binding, transphosphorylation of DAPK at a different site results in the activation of the kinase in a CaM-independent manner.

The second short talk, entitled “Nanoscale redistribution of NMDA receptor subunits in anti-NMDA receptor autoimmune encephalitis”, was presented by **Dr. Laurent Ladépêche** (Post-Doctoral researcher, Institut de Ciències Fotòniques (ICFO), Barcelona, Spain). Anti-*N*-Methyl-d-aspartate receptor (Anti-NMDAR) encephalitis is an autoimmune neuropsychiatric disease characterized by the disruption of neuronal synaptic antigens by patients’ auto-antibodies. The NMDAR is the main target of the immuno-response in the brain. To shed light on the molecular dynamics underlying its pathological internalization and depletion at a synaptic level, Dr. Ladépêche uses super resolution microscopy and computational simulations to study the effects of NMDAR auto-antibodies on the surface distribution of NMDA receptors on a nanoscale level. The results obtained in an in vitro neuronal system reveal that treatment with pathogenic antibodies results in early synaptic and extrasynaptic clustering and internalization of NMDARs, followed by redistribution of the remaining surface receptors, preferentially at a synaptic level. A comparison of the experimental results with Monte Carlo computational simulations allowed the group to model a system where pathological antibodies cross-link NMDAR receptors and leads to the disruption of NMDAR-protein interactions within and outside the synapse.

The next keynote lecture was given by **Prof. Elaine Fuchs** (Howard Hughes Medical Institute, Rockefeller University, New York, NY, USA). Her research is focused on understanding how stem cells transit between quiescence and proliferation and quiescence, a since this process that is likely to be involved in the origins of cancer. For this purpose, the group uses the hair follicle as a model system. Hair follicle stem cells remain quiescent for long periods but are able to start proliferating synchronously and cyclically for hair growth and wound repair. Understanding the biology of these cells in homeostatic conditions has shed light on the regulatory pathways controlling that balance and the interactions with neighboring “on the niche” cells required for such a transition. Prof. Fuchs’ team has applied these discoveries to squamous cell carcinomas (SCCs), one of the most common life-threatening cancers, and has shown the existence of a pool of tumor-initiating cells with stem cell characteristics, containing both active and quiescent cancer stem cells. The pool of active pool of stem cells dies upon cisplatin treatment, whereas the quiescent cancer stem cells receive high transforming growth factor-beta (TGF-β) from the nearby blood vessels. When the stem cells signal through TGF-β signaling, they undergo epithelial to mesenchymal transition-like transformation, and invade the surrounding tissue. This work has illuminated proven how the microenvironment influences stem cell biology and revealed the chromatin and transcriptional changes that underlie metastatic seeding of SCCs.

**Miriam Contreras** (PhD student, Department of Biochemistry and Molecular Biology, Faculty of Biology, Universitat de Barcelona, Barcelona, Spain) presented a short talk entitled “Definition and validation of targets associated with resistance-associated metabolic reprogramming in hematological malignancies”. Metabolic reprogramming is considered an adaptive behavior by which cancer cells elude and generate resistance to treatments. As a first step towards elucidating how the metabolism of Acute Myeloid Leukemia (AML) and Chronic Myeloid Leukemia (CML) cancer cells changes in response to therapy, her team generated resistant AML and CML cell lines through either treatment with chemotherapy drugs or by altering the expression of genes related to resistance acquisition. By comparing the metabolic differences between these cell lines and control cells sensitive to treatment, it will be possible not only to reveal metabolic changes through which cells adapt to cancer treatments, but also to identify potential metabolic targets to prevent the proliferation of resistant cells.

The last short talk of the session, entitled “Combining innovative genetic and imaging approaches to define mechanisms regulating aortic arch development”, was presented by **Ariadna Navarro-Aragall** (PhD Student, University College London (UCL), London, UK). Errors in the formation of the mammalian aortic arch from pharyngeal arch arteries (PAAs) lead to congenital heart defects—a major cause of perinatal mortality. A tightly regulated interaction between vascular endothelial cells and neural crest cells underlies this developmental process. By using genetic lineage tracing in mice, the researcher determined the role of the surface receptor Neuropilin 1 at various stages of PAA morphogenesis.

## 3. Conclusions and Future Perspectives of ENABLE

The first ENABLE conference was a success: 35 PhD and postdoc volunteers from four European research institutes, with support from the institute coordinators and Scienseed, organized an event in which 272 young researchers from over 25 countries presented and shared their science and experiences in scientific talks, poster sessions, master classes, general public talks, and evening activities. More than 60 attendees were given the opportunity to participate through the award of a travel grant funded by one of our 10 sponsors. The symposium, entitled “Breaking Down Complexity: Innovative Models and Techniques in Biomedicine”, was created to cover a broad range of topics in biomedical research, to encourage participation and the exchange of ideas, and to promote future collaborations among young scientists.

In order to include multiple research areas, there was a scientific program with four sessions. The first, entitled “Building the Foundations of Biology: Synthetic and Cellular Research”, featured Prof. Martin Hanczyc (University of Trento, Trento, Italy) and Prof. Elaine Fuchs (The Rockefeller University, New York, NY, USA) as keynote speakers and included short talks on DAPK regulation, nanoscale redistribution of NMDAR in autoimmune encephalitis, targets associated with metabolic reprogramming in hematological malignancies, and the mechanisms regulating aortic arch development. The second session, “The OMICS Revolution: Understanding the Layers of Life”, featured Prof. Johan Auwerx (Ecole Polytechnique, Lausanne, Switzerland) and Prof. Ruedi Aebersold (Eidgenössische Technische Hochschule (ETH), Zurich, Switzerland) as keynote speakers and included short talks on proteomics to study the role of polycomb repressive complex 2 (PRC2) in embryonic stem cells, single-cell sequencing to reconstruct the cell lineages of whole adult animals, the effects of endocrine-disrupting chemicals on development, and on quantitative proteomics to study fibrotic networks. The third session, “In Vitro to in Vivo: Modeling Life in 3D”, featured Prof. Kristina Havas Cavalletti (FIRC Institute of Molecular Oncology (IFOM), Milan, Italy) and Dr. Kim Jensen (Biotech Research and Innovation Centre (BRIC), Copenhagen, Denmark) as keynote speakers and included short talks on gut vascular barrier disruption and type 2 diabetes, the link between metabolic dysfunction and immune complications in lysinuric protein intolerance, the role of obesity in the development of acute promyelocytic leukemia, and the TGF-β pathway in colorectal cancer metastasis. The fourth and final session, “From Discovery to Cure: The Future of Therapeutics”, featured Prof. Eytan Ruppin (Univeristy of Maryland, Center for Bioinformatics and Computational Biology (CBCB), College Park, MD, USA) and Prof. Christian Brander (IrsiCaixa, Barcelona, Spain) as keynote speakers and included short talks on the role of miR27a as a tumor suppressor, photodynamic cancer therapy, real-time in vivo monitoring of transplanted islets, and a high-density lipoprotein nanodisc for the potential treatment of cerebral β-amyloidosis.

The success of the event was confirmed by the satisfaction scores (out of 5) given by the participants. In this regard, they gave the 2017 ENABLE symposium 4.4, the general topic of the symposium 4.1, and the keynote talks 4.4. The favorite part of the symposium was “Tapas with the Speakers” (score of 4.5), an activity that allowed the participants to interact with the keynote speakers in an informal setting while enjoying some typical Spanish food. The satisfaction of the attendees was also reflected by comments made on the evaluation form, such as “The option of travel grants is amazing and the general idea of the symposium is great. Great speakers, amazing food, nice event. Congratulations”, “I think that the ENABLE project is an amazing idea. It is a little bit different than other conferences because of the career day. The fact that it was organized by PhD students is really interesting!”, and “On the all, the symposia was super nice and it was extremely great to really discuss science on a reality level. No one wanted to show off or pretended to be the best scientist in the world and this was awesome”.

To conclude, the symposium brought together renowned scientists with young scientists and can be considered a huge success. The enthusiasm of the participants and the positive feedback received after the event underscore this notion and indicate that the ENABLE conference series has got off to an excellent start, with all eyes now focused on the 2018 event in Copenhagen.

The second symposium of the ENABLE series will be hosted by the Novo Nordisk Foundation Center for Protein Research (CPR, University of Copenhagen, Denmark), one of the partner institutions of the ENABLE consortium. It will take place 6–9 November 2018 at the Maersk Tower in Copenhagen.

Entitled “The Promise of Future Medicine: From Research to Therapy”, the symposium will explore state-of-the-art biomedical research from basic science to clinical practice and patient outcome. By bringing together 300 PhD students and postdocs, as well as nine eminent keynote speakers from diverse research fields, the next ENABLE symposium seeks to foster a multidisciplinary environment and crosstalk between biomedical disciplines. The following speakers have already confirmed their participation: Helen Lee (Cambridge University, Cambridge, UK); Giuseppe Testa (European Institute of Oncology, Milan, Italy); Nazneen Rahman (Institute of Cancer Research, London, UK); Klaus Pantel (Institute of Tumour Biology, University Medical Centre Hamburg-Eppendorf, Hamburg, Germany); Michel Morange (Institute for the History and Philosophy of Science and Technology (IHPST), Paris, France); Andrea Bertotti (Istituto di Ricovero e Cura a Carattere Scientifico (IRCCS), Candiolo, Italy); and Matthew Wood (University of Oxford, Oxford, UK).

Apart from the scientific symposium, a Career Day is foreseen, to allow participants to broaden their career perspectives. This activity will involve chats with professionals, high-quality workshops, and an Opportunity Fair, which will allow participants to come into direct contact with companies belonging to a variety of sectors. To support the participation of young researchers from all over Europe, our sponsors will provide about 40 travel grants to cover the registration fee and travel and accommodation expenses. Up-to-date information on the event can be found on our website (https://enablenetwork.eu/).

We look forward to the 2018 symposium and are confident that ENABLE will foster the establishment of a network that promotes efficient and synergistic scientific exchange among researchers throughout Europe.

